# Insulin Resistance and Endothelial Dysfunction Constitute a Common Therapeutic Target in Cardiometabolic Disorders

**DOI:** 10.1155/2016/3634948

**Published:** 2016-06-20

**Authors:** A. Janus, E. Szahidewicz-Krupska, G. Mazur, A. Doroszko

**Affiliations:** ^1^Department of Internal Medicine, Occupational Diseases Hypertension and Clinical Oncology, Wroclaw Medical University, Ulica Borowska 213, 50-556 Wrocław, Poland; ^2^Research and Development Department, Integrated Cardiovascular Centre Provincial Specialist Hospital in Wroclaw (WROVASC), Ulica Kamieńskiego 73a, 51-124 Wrocław, Poland

## Abstract

Insulin resistance and other risk factors for atherosclerosis, such as hypertension and hypercholesterolemia, promote endothelial dysfunction and lead to development of metabolic syndrome which constitutes an introduction to cardiovascular disease. The insulin resistance and endothelial dysfunction cross talk between each other by numerous metabolic pathways. Hence, targeting one of these pathologies with pleiotropic treatment exerts beneficial effect on another one. Combined and expletive treatment of hypertension, lipid disorders, and insulin resistance with nonpharmacological interventions and conventional pharmacotherapy may inhibit the transformation of metabolic disturbances to fully developed cardiovascular disease. This paper summarises the common therapeutic targets for insulin resistance, endothelial dysfunction, and vascular inflammatory reaction at molecular level and analyses the potential pleiotropic effects of drugs used currently in management of cardiovascular disease, metabolic syndrome, and diabetes.

## 1. Introduction

Insulin plays an important role in maintenance of vascular homeostasis. On one hand insulin stimulates endothelial production of nitric oxide (NO), a crucial vasodilator exerting an antiaggregatory effect and limiting vascular smooth muscle cells growth and migration, but on the other one mediates the release of endothelin ET-1, known to act as a strong vasoconstrictor [1]. This dual action of insulin is mediated by two major signalling pathways. Under physiological conditions, a vasoprotective phosphoinositide-3-kinase (PI3-K)/Akt pathway predominates and is responsible for expression and activation of endothelial nitric oxide synthase (eNOS) [2].

When insulin resistance appears, the balance is shifted towards mitogen-activated protein kinase/extracellular signal-regulated kinase (MAPK/ERK), which mediates inflammation, vasoconstriction, and vascular smooth muscle cell proliferation [3]. The crosstalk between insulin signalling pathways and endothelial metabolism is strongly related. Therefore, insulin resistance commonly coexists with endothelial dysfunction in cardiovascular disease. Both nonpharmacological and pharmacological interventions act on amelioration of insulin sensitivity as well as on improvement in endothelial function [4].

## 2. Insulin Signalling (Figure 1)

Insulin binds to insulin receptor IR, which contains the two *α* and two *β* subunits. The *α* subunit binds insulin, insulin growth factor-1 (IGF-1), and epidermal growth factor (EGF). The *β* subunit contains extracellular, transmembrane, and cytosolic domains. The cytosolic part of the *β* subunit has tyrosine kinase activity, which undergoes conformational changes and is autophosphorylated after insulin biding to the *α* subunit. Activated IR phosphorylates also number of proteins on tyrosine residues, for example, insulin receptor substrate (IRS), Shc proteins, or Gap-1 [5]. In human cells three isoforms of IRS (IRS-1, -2, and -4) were identified to play a distinct role, depending on cell type and metabolic state. Also those two insulin receptor substrates represent different kinetics, compartment distribution, and substrate interactions (IRS-1 is a transmembrane protein and IRS-2 is mostly present in cytosol) [6]. IRS-1 plays a crucial role in skeletal muscle and its function is to provide insulin secretion mechanisms [7]. IRS-2 is responsible for insulin action in liver and pancreatic *β* cells development. Animal models showed that IRS-1 knockout mice had growth retardation especially in skeletal muscle and liver, but not in brain [8]. Mice lacking IRS-1 developed insulin resistance with hyperinsulinemia, not diabetes, but displayed features of metabolic syndrome (hypertension and hypertriglyceridaemia) [8]. Animals without IRS-2 exhibited insulin resistance with fasting hyperglycemia, due to inadequate insulin production, which in final resulted in diabetes, which was worse than lack of IRS-1 [8]. IRS tyrosine phosphorylation is mandatory for insulin response, but depending on which serine is phosphorylated, IRS intensifies or diminishes insulin action [9].

## 3. The PI3-K/Akt Pathway

The phosphorylation of IRS tyrosine activates phosphoinositide-3 kinase (PI-3K), which converts phosphatidylinositol (3,4)-bisphosphate (PIP2) to a second messenger phosphatidylinositol (3,4,5)-trisphosphate (PIP3) [10]. PIP3 facilitates translocation of Akt kinase from inactivated form to the cell membrane, where is activated by phosphoinositide-dependent kinase-1 (PDK-1) [11]. The Akt activation on Thr308 and Ser473 has many implications in cellular processes. Except for cell survival, growth, and proliferation, Akt influences also glucose metabolism, nitric oxide production, and angiogenesis [12]. In endothelial cells Akt activation may induce undesirable proliferation and survival of tumour vasculature [13], but in insulin resistant state diminished cell proliferation may lead to atherosclerosis, decreased collateral angiogenesis in occluded coronary and lower extremities vasculature, or reduced reendothelialisation [14]. The antiapoptotic effect of Akt phosphorylation is mediated by inhibition of caspase-9, which prevents endothelial cells from death induced by inflammatory response [15]. The crosstalk between endothelial cells and insulin signalling pathway is marked also in Akt phosphorylation at Ser1177 of endothelial nitric oxide synthase (eNOS) [2], which enhances antiapoptotic effect in ischemic myocardium and stimulates vasodilation and angiogenesis by nitric oxide production [16]. The eNOS activation is mediated by inhibition of calmodulin dissociation and electron transfer in a reductase domain [17]. Proangiogenic role of Akt is expressed by increased production of the hypoxia-inducible factor *α* (HIF1*α* and HIF2*α*), which leads to secretion of proangiogenic factors for example vascular endothelial growth factor (VEGF) [18].

## 4. The MAPK/ERK Pathway 

The MAPK pathway is activated by insulin, which results in cytosolic growth factor receptor-bound protein 2 (Grb2) binding to the plasma membrane. Grb2 interacts with IRS by Src homology and collagen protein (Shc). Grb2 is also associated with proline-rich domain of the son of sevenless (SOS), which is the guanyl nucleotide-exchange factor. This process triggers transformation of inactive GDP-bound Ras into active form of GTP-bound Ras [19]. Active Ras stimulates serine/threonine kinase Raf, which phosphorylates and activates MEK1/2. MEK1/2 phosphorylate in turn ERK, a member of the MAPK signalling enzymes [20]. MAPK pathway is also associated with endothelial cells by mediating secretion of ET-1 [21].

## 5. Insulin Resistance (Figures 2 and 3)

Insulin resistance refers to the state of decreased insulin response and is a common feature of obesity, hypertension, diabetes, and coronary artery disease [22]. Impairment of PI3-K/Akt signalling pathway leads to an inadequate tissue insulin sensitivity. The paradox of pathologies in molecular insulin signalling contributes to diminished activity of the PI3-K/Akt pathway coexisting with strengthened MAPK/ERK pathway, during compensatory hyperinsulinaemia [23]. Differences in activity of both pathways are responsible for divergences in insulin resistance in different organs for example lack of suppression of glucose production by insulin and maintained lipogenesis in the liver [24] or decreased production of nitric oxide and enhanced production of ET-1 in endothelium [25]. Insulin resistance is associated inseparably with glucotoxicity, lipotoxicity, and inflammation, which initiates and accelerates atherogenesis and vascular disease [26].

Changes in balance between the PI3-K/Akt and MAPK/ERK pathways provide strong relationship between insulin resistance and endothelial dysfunction [27]. What is more, when the balance in insulin resistance is shifted towards the MAPK/ERK pathway, it results in a release of inflammatory markers by insulin (e.g., PAI-1, ICAM-1, VCAM-1, and E-selectin) and finally promotes the endothelial dysfunction [28].

## 6. Endothelial Dysfunction (Figures 2 and 3)

Endothelium is a multifunctional paracrine, autocrine, and endocrine organ, “the ranger” of vascular homeostasis. The endothelial balance is maintained by substances of vasodilatory action (e.g., NO or prostaglandins (PGI2)) and vasoconstricting features (e.g., angiotensin II (Ang II) or ET-1) [29]. Insulin, by acting through distinct metabolic pathways, may influence both groups of factors. Activation of the PI3-K/Akt pathway leads to phosphorylation of eNOS and subsequent conversion of L-arginine to L-citrulline and NO, the most important vasodilator. NO plays also protective role for endothelium by decreasing expression of cell adhesion molecules, attenuating platelet aggregation, production of proinflammatory cytokines, and inhibiting vascular smooth muscle cells proliferation [30]. Deficiency in the NO bioavailability, increased level of prothrombotic and proinflammatory markers, and reactive oxygen species (ROS) are factors indicating endothelial dysfunction, which are mediated by MAPK/ERK activity. Glucotoxicity and lipotoxicity generate inflammatory reaction contributing to vascular damage and link insulin resistance with endothelial dysfunction through different mechanisms.

## 7. Glucotoxicity in Insulin Resistance and Endothelial Dysfunction

Hyperglycemia activates the hexosamine biosynthesis pathway and modifies proteins involved in insulin and NO signalling by the O-Glc-N-acylation of IRS-1, which impairs activation of PI3-K and reduces glucose uptake [31] and O-Glc-N-acylation of eNOS at the Akt phosphorylation residues, leading to its inactivation [32]. O-Glc-N-acylation also induces PAI-1 gene expression and alters tumor growth factor *β* (TGF*β*) level, what is related to pathogenesis of vascular diabetic damage [33, 34]. The overactivation of hexosamine biosynthesis pathway results in formation of advanced glycation end products (AGEs), which in turn stimulate ROS production. Reactive carbonyl species (RCS) are formed in the course of oxidation of carbohydrates, lipids, and amino acids and have been identified as intermediates in the formation of irreversible, advanced glycoxidation and lipoxidation end products (AGEs and ALEs) on protein. Reactive carbonyl, oxygen, and nitrogen species (RCS, ROS, and RNS, resp.) are now recognized to be important transducers in biological systems. There is a growing body of population of structurally defined AGE products such as pyrraline, pentosidine, N-carboxy-methyl lysine (CML), and crossline that are found to be elevated in diabetic tissues. Some of the highest levels of pentosidine have been detected in individuals with diabetes. There is also some evidence for elevated skin pentosidine levels in individuals with diabetes correlate with the severity of the complications [35–37].

Increased oxidative stress enhances insulin resistance by impairing Akt and eNOS activation and limiting NO availability [38]. Moreover, ROS stimulates IKK*β* kinase, which leads to activation of NF-*κ*B and overexpression of proinflammatory markers, for example, interleukin-1*β* (IL-1*β*), tumour necrosis factor-*α* (TNF-*α*), and phosphorylation and inactivation of IRS-1 [39]. ROS forming oxidant peroxynitrites (ONOO^−^) enhance endothelial dysfunction by direct uncoupling and inactivating the eNOS.

Modification of endothelial cells matrix collagen and laminin by AGEs impairs vascular elasticity and interaction with macrophages promotes atherosclerosis [40]. Vascular remodelling of vessels associated with cardiometabolic disorders seems to be hypertrophic and it is mostly due to increased extracellular matrix deposition. The mechanisms underlying the obesity-, insulin resistance-, and/or hyperinsulinemia-induced vascular disease are not fully understood but might include hemodynamic factors such as hypertension, activation of the renin-angiotensin-aldosterone system, metabolic factors such as insulin and advanced glycation end products, and other factors such as adipokines, inflammation, or oxidative stress [41]. Hyperglycemia promotes AGEs production, which inhibit tyrosine phosphorylation of IRS-1 and IRS-2 and decrease activation of the PI3-K/Akt pathway by activation of phosphokinase C (PKC) [42]. Adipose tissue has been demonstrated to be an active organ, where matrix metalloproteinases (MMPs) play an important role in adipogenesis, angiogenesis, and proliferation of extracellular matrix. However, the lack of association between adipose tissue and plasma levels of some MMPs, specifically MMP-2 and MMP-9, suggests that this tissue is not a major contributor to circulating MMPs. These enzymes, which are responsible for tissue remodelling, are also expressed in response to inflammatory adipocytokines, like adiponectin or leptin. Adiponectin may also play a protective role in the plaque rupture through selectively increasing the tissue inhibitor of metalloproteinase (TIMP) expression. Leptin induces expression of MMP-2 activators and the expression of MMP-2, MMP-9, and TIMP-1 in numerous human cells [43].

## 8. Lipotoxicity in Insulin Resistance and Endothelial Disfunction

Lipotoxicity inhibits the PI3-K/Akt signalling and activates the MAPK/ERK pathway by inducing oxidative stress and inflammation through free fatty acids (FFA) [44]. FFA stimulate PKC impairing Akt function due to IRS 1/2 inactivation [45] and enhance NADPH oxidase to ROS production [46]. NADPH oxidase induces production of PAI-1, interleukin-6 (IL-6), and chemokine (C-C motif) ligand 2 (CCL-2), which increase proinflammatory state and inhibits NO production by decreasing eNOS expression. Moreover, ROS after FFA stimulation activate NF-*κ*B, which increases ET-1 expression and adhesion molecules ICAM-1 and VCAM-1 and increase cardiovascular risk even in healthy subjects [47].

The adhesion molecules on endothelial cells promote their contact with monocytes, which turn into macrophages absorbing lipoproteins and as the foam cells secrete IL-6 and TNF-*α*. TNF-*α* and IL-6 mobilize immune cells to build atherosclerotic plaque and activate IKK*β*, which leads to impaired insulin signalling in endothelial cells and activates NF-*κ*B [48].

## 9. Nonpharmacological Interventions Improving Insulin Sensitivity and Endothelial Function

An imbalance between the PI3-K/Akt and MAPK/ERK pathways links insulin resistance and endothelial dysfunction. Pathology of decreased activation of Akt signalling with diminished NO production and stimulation of MAPK pathway is commonly contributed to overweight, obesity, and diabetes [49]. Dietary intervention leading to restoration of the balance between both pathways targets insulin sensitivity and endothelial function. There are animal and human studies demonstrating beneficial effect of polyphenols added to diet, based on their involvement in pathways described above. Green tea polyphenol (EGCG) has been discovered to mimic insulin action via PI3-K pathway, by stimulating glucose uptake and inhibiting hepatocyte gluconeogenesis [50]. Moreover, EGCG is involved in pathway regulating eNOS activation and NO production in endothelium [51]. This feature of green tea polyphenol contributes to its antidiabetic, insulin sensitizing, and lipid lowering properties [52]. Another floral eatable polyphenol of eNOS activating effect is hesperidin, extracted from citrus fruit. Hesperidin is shown to reduce the triacilgliceroles level and free fatty acid oxidation with decrease in inflammatory markers [53]. Cocoa flavonoids also showed positive effect on eNOS activity and endothelial improvement as well as on insulin sensitivity in several short-term studies [54]. Animal studies involving dietary restriction of AGEs elimination also revealed satisfying effect on metabolic disturbances. Reversing insulin resistance combined with suppressing the inflammation and atherosclerosis might be a future therapeutic option [55]. AGEs are absorbed from highly heated processed food (barbeque, grilled) and higher levels were shown to correspond with vascular damage [56]. However, low-quality evidence of human studies needs further investigation [57].

Meta-analyses, which compared different dietary patterns, have shown that the Mediterranean diet has beneficial effect on cardiovascular disorders, cancer risk [58], and diabetes [59]. Nonpharmacological interventions combine also diet with physical exercise, which is demonstrated to reduce inflammatory markers and improve insulin sensitivity [60]. Lifestyle modifications can stop and reverse disease, which was shown by Esposito et al. by comparing Mediterranean to low-fat diet with an effect of remission of diabetes and delay of drug requirement [61].

## 10. Pharmacological Interventions Improving Insulin Sensitivity and Endothelial Function (Figure 2)

### 10.1. Thiazolidinediones

Thiazolidinediones bind to peroxisome proliferator-activated receptor (PPAR-*γ*). PPAR-*γ* regulates transcription of insulin sensitive genes, which control glucose and lipid metabolism. Thiazolidinediones improve insulin sensitivity and decrease FFA circulating amounts [62]. Their anti-inflammatory properties are expressed by decrease in expression of adhesion molecules, ICAM-1, VCAM-1, and E-selectin, which protect monocytes from vascular wall attachment and later lipid accumulation in macrophages [63]. PPAR-*γ* ligands inhibit NF*κ*B and decrease inflammation that way. Thiazolidinediones inhibit NADPH oxidase expression components NOX1, NOX2, and NOX4, reduce ROS production, increase NO formation through heat shock protein 90 and eNOS interaction [64], promoting vasodilation, and suppress ET-1, protecting from vasoconstriction. PPAR-*γ* ligands decrease vasculature complications in diabetes, by lowering fasting insulin level and blood pressure, and reduce secondary clinical end point of stroke and myocardial infarction death.

### 10.2. Dimethylbiguanide

Metformin is an oral first-line treatment in diabetes 2 and is not associated with a hypoglycemic tendency. Metformin exerts its antihyperglycemic effect by decreasing hepatic glucose production by suppressing of gluconeogenesis and enhancing insulin suppression of endogenous glucose production, by reducing intestinal glucose reabsorption and possibly improving glucose uptake and utilization by peripheral tissues, such as skeletal muscle, and adipose tissue lowers blood glucose levels [65]. It acts via the AMP-activated protein kinase (AMPK) and by eNOS phosphorylation and NO increased production ameliorates endothelial function [66]. Despite AMPK pathway, metformin inhibits the respiratory chain complex-1 (NADH:ubiquinone oxidoreductase) in mitochondria [67] and regulates oxidative stress induced by hyperglycemia. Metformin plays also a crucial role in the incretin pathway through the glucagon-like peptide (GLP-1), by enhancing its production [68].

### 10.3. Glucagon-Like Peptide-1 Connected Drugs

GLP-1 is a hormone generated and secreted from enteroendocrine cells of intestine, which enhances glucose-stimulated insulin secretion and suppresses glucagon release thereby modulating both postprandial and long-term glucose homeostasis [69]. It acts through the G-protein coupled receptor (GLP-1R). GLP-1 is inactivated by the serine protease dipeptidyl-peptidase-4 (DPP-4) [70]. Soluble form of dipeptidyl-peptidase-4, which is present in plasma, is inactive against novel diabetic drugs degradation-insensitive GLP-1R agonists (exenatide, liraglutide, and lixisenatide). Liraglutide (NN2211) is a long-acting glucagon-like peptide-1 receptor agonist, binding to the same receptors as does the endogenous metabolic hormone GLP-1 that stimulates insulin secretion. Exenatide (NN2211) is a 39-amino acid peptide, an insulin secretagogue, with glucoregulatory effects, and is a long-acting glucagon-like peptide-1 receptor agonist, binding to the same receptors as does the endogenous metabolic hormone GLP-1 that stimulates insulin secretion. Lixisenatide has been described as “des-38-proline-exendin-4 (*Heloderma suspectum*)-(1–39)-peptidylpenta-L-lysyl-L-lysinamide,” meaning it is derived from the first 39 amino acids in the sequence of the peptide exendin-4, found in the Gila monster (*Heloderma suspectum*), omitting proline at position 38 and adding six lysine residues [71].

GLP-1 has vasoprotective properties, including its effects on heart rate, ischemia/reperfusion injury, coagulation, inflammation, and vascular endothelial function [72]. GLP-1 agonists reveal vasodilatory properties, by increasing the NO production, stimulating proliferation, and protecting from lipid-induced apoptosis of human endothelial cells, through PI3K/Akt pathway, protein kinase A (PKA), and the eNOS-dependent pathways [73]. Liraglutide reduces inflammatory cytokine (TNF-*α*) and hyperglycemia-induced expression of the fibrinolysis inhibitor, PAI-1, and vascular adhesion molecules VCAM-1 and ICAM-1, which decreases inflammation and monocytes attachment [74]. In animal models GLP-1 agonist diminished monocyte adhesion, macrophage infiltration, and atherosclerotic lesions in the vasculature [75].

High activity of the DPP-4 enzyme in immune system might give a possibility of using dipeptidyl peptidase-4 inhibitors in anti-inflammatory therapy, particularly in atherosclerosis. DPP-4 inhibitors mediate macrophages polarization in atherosclerotic regions, decrease the level of M1 macrophages, responsible for inflammation [76], and expand anti-inflammatory M2 macrophages, which, in turn, might diminish insulin resistance and ameliorate endothelial function. Inflammatory reactions might be reduced by GLP-1R agonists and DPP-4 inhibitors, due to macrophages shift into M2 type through T regulatory lymphocytes (Tregs), whose function is increased by GLP-1 [77]. Moreover, Tregs secrete interleukin-10 (IL-10), which inhibits NADPH oxidase, reducing oxidative stress and ROS production. This additional metabolic role protects endothelium and maintains correct insulin signalling, since NADPH oxidase has been shown to activate serine kinases, which phosphorylate IRS and disrupt physiological insulin pathway [78]. Pharmacological inhibition of dipeptidyl peptidase-4 increase the bioavailability of GLP-1, which enhances insulin-dependent action in vasculature. Saxagliptin (rINN), previously identified as BMS-477118, linagliptin (BI-1356), vildagliptin (LAF237), sitagliptin (MK-0431), and alogliptin are oral hypoglycemic agent of the dipeptidyl peptidase-4 (DPP-4) inhibitor class of drugs approved by the FDA for management of type 2 diabetes in adults. Animal studies of obese Zucker rats treated with linagliptin showed improvement in eNOS activation, blood pressure, and diastolic heart function [79]. Nonetheless, two large clinical studies with DPP-4 inhibitors, EXAMINE [80], which involved alogliptin, and SAVOR-TIMI 53 [81], which involved saxagliptin, did not show reduced risk of cardiovascular events, but further investigations are needed [82].

## 11. Drugs Acting on the Renin-Angiotensin-Aldosterone System 

In insulin resistance and endothelial dysfunction, a hyperactivity of the renin-angiotensin-aldosterone system (RAAS) plays a crucial role, and therefore targeting it on a different molecular level benefits in improvement in insulin sensitivity and vascular function. The most harmful factor in this system, affecting insulin metabolism and endothelium, is angiotensin II (Ang II). Ang II is converted from inactive angiotensin I by the angiotensin converting enzyme (ACE) and acts as a ligand for angiotensin II receptors, mostly type 1 (AT1). Angiotensin II interferes with the insulin pathways by suppressing IRS-1 phosphorylation and decreasing PI3-K function and glucose receptor (GLUT-4) translocation, which diminishes glucose uptake [83]. Moreover Ang II hinders endothelial function by decreasing NO bioavailability through NADPH oxidase activation and ROS production. Destructive function of Ang II affects also endothelium by enhancing NF-*κ*B, which in turn promotes production of TNF*α* and IL-6 and adhesion molecule VCAM-1, mediating inflammation [84]. Association between RAAS, insulin, and endothelial pathways results in wide use of drugs targeting those common pathologies, and therefore the treatment with ACE inhibitors, which reduce circulating AngII levels and angiotensin receptor blockers (ARBs), has additional benefits beyond antihypertensive effect. This metabolic outcome results from blocking the crosstalk between Ang II and insulin at the level of IRS-1 and PI3-K [85]. Human and animal studies showed that ACE inhibitors and ARBs have positive effect on glucose disposal in glucose intolerance, diabetes mellitus, obesity, and hypertension [86]. In line with these reports, some trials have shown that ACE inhibitors and ARBs improve insulin sensitivity and prevent new onset of diabetes [87]. In the DREAM trial (Diabetes Reduction Assessment with Ramipril and Rosiglitazone Medication) ramipril reduced the postchallenge glucose levels and increased the tendency of regression to normoglycemia in subjects with impaired glucose tolerance and impaired plasma glucose levels [88]. In the TREND study (Trial on Reversing Endothelial Dysfunction) another ACE inhibitor, quinapril, has been shown to improve endothelial function by enhancement in the NO release in normotensive subjects with coronary artery disease [89]. Increase in NO production might facilitate glucose delivery to tissues due to vasodilation. ARB representative, losartan, also increased insulin sensitivity, improved endothelial function, and impacted inflammatory markers in hypercholesterolemic hypertensive patients [90]. Different mechanisms of crosstalk between insulin and endothelial pathways are perfectly optimized during telmisartan treatment, due to its dual action, which consists of angiotensin receptor blockade and activation of the peroxisome proliferator-activated receptor-*γ* (PPAR-gamma) [91].

## 12. Hypolipemic Drugs

In pathologies accompanied by hyperlipidemia, the two types of therapeutic regimens are commonly used: the 3-hydroxy-3-methylglutaryl-CoA reductase inhibitors (statins) and fibrates. Statins are characterized by improving endothelial function, reducing inflammation and ET-1 circulating levels, which diminishes vasoconstriction and ameliorates the insulin activity [92], especially in addition to ACE inhibitors or ARBs. Fibrates act as a PPAR-*α* ligands improving lipid profile, insulin sensitivity, and endothelial function and diminishing vascular inflammation, which has been shown in the FIELD study [93].

## 13. Conclusions

Endothelial and insulin signalling pathways crosstalk each other and therefore the relationship between endothelial function and insulin metabolism is very important in disorders, such as hypertension, obesity, or diabetes. Insulin resistance, a hallmark of metabolic syndrome, impairs vascular response and increases cardiovascular risk. Involvement of insulin resistance and endothelial dysfunction in pathological disorders contribute to impairment in the NO-dependent vasodilatation, cellular glucose uptake, enhancement in oxidative stress, and inflammation, leading finally to atherosclerosis. Strong association of insulin and endothelial signalling disturbances contributes to glucotoxicity, lipotoxicity, and inflammation, disrupting the balance between vasodilating-vasoconstrictive endothelial mechanisms as well as between the insulin-dependent PI3-K/Akt–MAPK/ERK pathways. The synergistic antidiabetic, antihypertensive, and hypolipemizing treatment, aiming at multiple metabolic pathways, improve both insulin sensitivity and endothelial function and should be considered at early stages of disturbances, before clinical progression of diseases, with fully developed vascular complications.

## Figures and Tables

**Figure 1:**
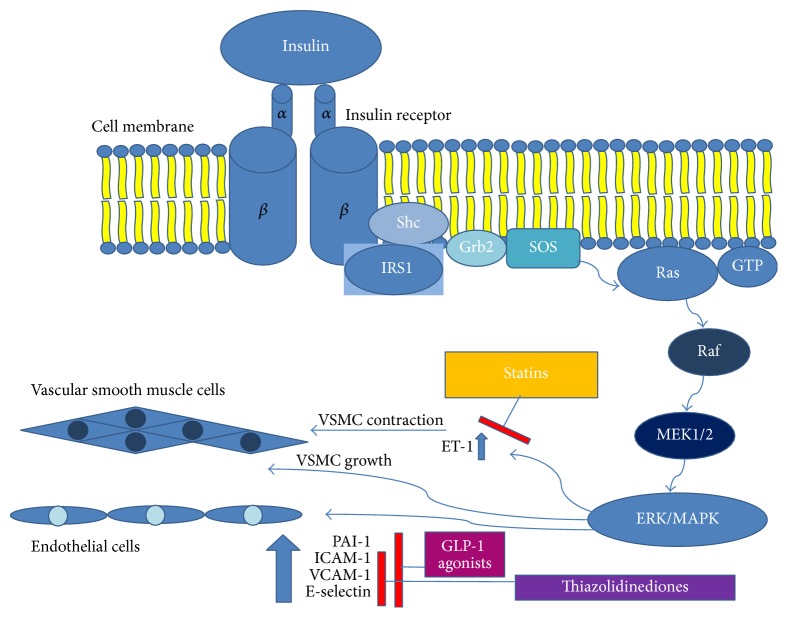
Insulin signalling in vessels. *α*: alfa subunit of insulin receptor; *β*: beta subunit of insulin receptor; Shc: Src homology and collagen protein; Grb2: cytosolic growth factor receptor-bound protein 2; IRS1: insulin receptor substrate; SOS: proline-rich domain of the son of sevenless; Ras: family of related proteins; Raf: serine/threonine specific protein kinases; MEK1/2: mitogen-activated protein kinase; ERK/MAPK: mitogen-activated protein kinase; ET-1: endothelin-1; GLP-1: glucagon-like peptide; PAI-1: plasminogen activator inhibitor; ICAM-1: intercellular adhesion molecule 1; VCAM-1: vascular cell adhesion molecule 1.

**Figure 2:**
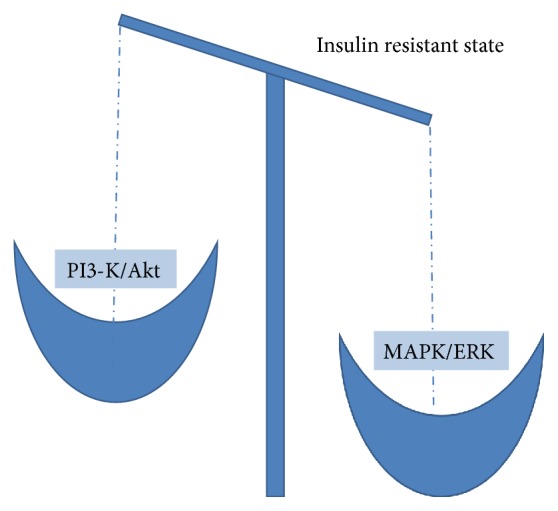
Conceptual definition of insulin resistance at molecular level.

**Figure 3:**
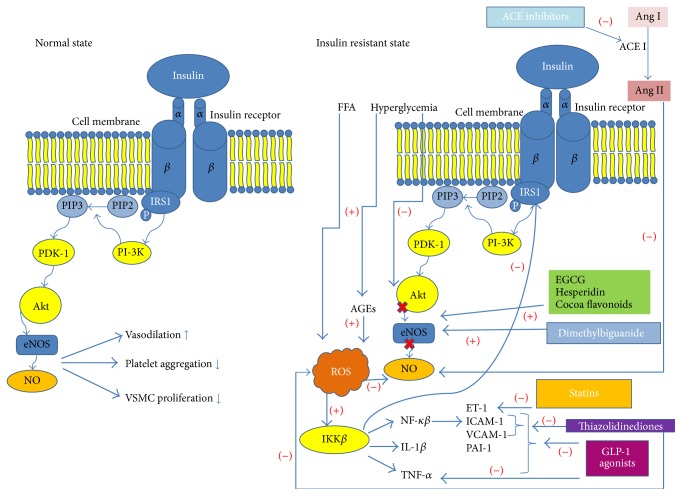
Changes in expression of particular molecular pathways in insulin resistant state versus under physiological condition. Molecular definition of drug targets for management of insulin resistance. *α*: alfa subunit of insulin receptor; *β*: beta subunit of insulin receptor; Shc: Src homology and collagen protein; Grb2: cytosolic growth factor receptor-bound protein 2; IRS1: insulin receptor substrate; SOS: proline-rich domain of the son of sevenless; Ras: family of related proteins; Raf: serine/threonine specific protein kinases; MEK1/2: mitogen-activated protein kinase; ERK/MAPK: mitogen-activated protein kinase; ET-1: endothelin-1; GLP-1: glucagon-like peptide; PAI-1: plasminogen activator inhibitor; ICAM-1: intercellular adhesion molecule 1; VCAM-1: vascular cell adhesion molecule 1; Ang I: angiotensin I; Ang II: angiotensin II; ACE I: angiotensin converting enzyme I; TNF-alpha: tumour necrosis factor alpha; ROS: reactive oxygen species; Akt: the Akt kinase; eNOS: endothelial nitric oxide synthase; AGEs: advanced glycation end products.
